# Personality and substance use disorder: Characteristics as measured by NEO-personality inventory–revised

**DOI:** 10.3389/fpsyg.2022.982763

**Published:** 2022-11-07

**Authors:** Elise Constance Fodstad, Anastasia Ushakova, Ståle Pallesen, Egon Hagen, Aleksander Hagen Erga, Eilin Kristine Erevik

**Affiliations:** ^1^Department of Psychosocial Science, Faculty of Psychology, University of Bergen, Bergen, Norway; ^2^Centre for Alcohol and Drug Research (KORFOR), Stavanger University Hospital, Stavanger, Norway; ^3^Department of Research, Section of Biostatistics, Stavanger University Hospital, Stavanger, Norway; ^4^Department of Social Studies, Faculty of Social Sciences, University of Stavanger, Stavanger, Norway

**Keywords:** NEO-PI-R, five factor model, Big Five, addiction, polysubstance use

## Abstract

The present study investigates the personality characteristics of a cohort of patients with Substance Use Disorders. The included participants (*n* = 123) were recruited from specialized treatment for addictions in Norway. The personality scores in the current sample were compared to the Norwegian norm sample with *t*-tests. Age and gender differences in personality scores were assessed by bivariate correlation analyses and *t*-tests, respectively. The sample had higher scores on Neuroticism and lower scores on Conscientiousness, Agreeableness, Extraversion, and Openness compared to the norm sample (*p* < 0.01). The effect sizes of the differences between the current sample and the Norwegian norm sample were large for Neuroticism and Conscientiousness. Older participants scored higher on Agreeableness and its facets A1: Trust and A2: Straightforwardness and lower on the facet E5: Excitement-Seeking (*p* < 0.01). No significant (*p* < 0.01) gender differences in NEO-PI-R scores were found. In conclusion, the current results support previous findings regarding personality traits associated with SUD. The clinical relevance of the findings is discussed.

## Introduction

The Five-Factor Model of personality (FFM) is the most widely used and acknowledged taxonomy of personality traits (McCrae and John, 1992; Larsen et al., 2017). FFM was developed based on a lexical approach where trait adjectives in particular English languages were analyzed, and through questionnaires and statistical approaches such as factor analysis (McCrae and John, 1992; McCrae and Costa, 2010). FFM includes variants of the five broad traits (the *Big Five*), commonly known as Extraversion, Agreeableness, Conscientiousness, Neuroticism, and Openness (Goldberg, 1990). Measures of the FFM have been translated into several languages, and the results indicate that the traits reflect essential individual differences in different cultures (De Fruyt et al., 2009; Fedvadjiev and van de Vijever, 2015). Hence, the five traits have been suggested to have biological and evolutionary underpinnings (McCrae et al., 1998; Buss, 2009).

The NEO Personality Inventory–Revised (NEO-PI-R) is one of the most widely used instruments to assess the FFM (Costa and McCrae, 1995). In the NEO-PI-R, each of the broad, global five traits are conceptualized to have six underlying facets. An example is Extraversion, which consists of the six facets *E1: Warmth, E2: Gregariousness, E3: Assertiveness, E4: Activity, E5: Excitement-Seeking*, and *E6: Positive Emotions*. The NEO-PI-R has been translated to several languages and thoroughly investigated in different populations (Bagby et al., 1999; Caprara et al., 2001; Carter et al., 2001; Aluja et al., 2005; McCrae and Costa, 2010; Kallmen et al., 2011), including a Norwegian translation and norming (Martinsen et al., 2003, 2011; Martinsen, 2017) and a validation in an American Substance Use Disorder (SUD)-cohort (Piedmont and Ciarrocchi, 1999). These validations have found satisfactory internal consistency between the different items of the five traits and the facets, stable factor loadings and high test-retest reliability, as well as good content and criterion validity (see the manual chapter 8 for details; McCrae and Costa, 2010). Traits in the FFM correlate, and it is suggested that the five traits sort into two overarching meta-traits; *Stability* and *Plasticity*. *Stability* (or *beta*) reflects the shared variance of Neuroticism (inversely associated), Agreeableness and Conscientiousness, while *Plasticity* (or *alfa*) includes the shared variance of Extraversion and Openness (DeYoung and Allen, 2019). Overarching these two meta-traits, *Big One* has been suggested as a general factor of personality in which scores on Agreeableness, Conscientiousness, Extraversion and Openness are positively associated and inversely associated with Neuroticism (Musek, 2007).

Substance use disorders (SUDs) are associated with adverse health outcomes and shortened life expectancy (Whiteford et al., 2015), and is characterized by a loss of control over substance use over time. During recovery from SUDs, relapse is common (Bradizza et al., 2006). Personality could be one aspect influencing health outcomes and recovery from SUD (Ciraulo et al., 2003; Brorson et al., 2013). Knowledge about personality and SUD might help personalize treatment, i.e., if subgroups of SUD tend to have more deviant personality traits that will influence treatment compliance. Norwegian patients with SUD have shown high comorbidity with personality disorder symptoms (Korsgaard et al., 2016; Arnevik et al., 2019). The diagnostics of personality disorders are changing from categorical labels to dysfunctional traits corresponding to extremes of the FFM (American Psychiatric Association, 2013; Bach and First, 2018; World Health Organization, 2019; Widiger, 2020). Hence, expanded knowledge about typical personality traits among patients with SUD might inform which personality disorders might be the most common among individuals with SUD according to the new diagnostic frameworks of personality disorders. If patients with SUD, known to have high comorbidity of personality disorders, do not differ that much from the norms in their personality profile, this challenges the dimensional view of personality disorders as extremes of the FFM.

Patients with SUD tend to score high on Neuroticism, and low on Conscientiousness and Agreeableness compared to individuals without SUD across different measures of FFM (Kotov et al., 2010; Delic et al., 2017; Raketic et al., 2017). This SUD-profile is equivalent to a low score on the meta-trait *Stability* (DeYoung and Allen, 2019). Kotov et al. (2010) found that several psychiatric disorders were linked to high Neuroticism. Neuroticism is essentially related to the frequency and intensity of negative affect, e.g., anxiety, tension, and worry (McCrae and John, 1992). It follows logically that persons high on this trait will have a lower threshold for suffering from psychiatric disorders. In addition, the use of substances and a life of substance use often cause psychological distress, which might contribute to higher scores on Neuroticism. Conscientiousness, a trait that captures the tendency to plan and organize (McCrae and John, 1992), is also linked to several psychiatric disorders (Kotov et al., 2010). Psychological distress might drain psychological resources to plan and organize or people with low Conscientiousness might be more vulnerable to developing psychiatric disorders following stressful life events. Furthermore, Conscientiousness has phenomenological overlap with executive functioning, which is reduced in patients with SUD (Hagen et al., 2016). The overlap between Conscientiousness and executive functioning, control mechanisms regulating cognition and behavior, and which concept is more useful to predict health are being discussed (Bogg and Roberts, 2013). A broader knowledge of which facets of Conscientiousness diverge in different subgroups of SUD might inform this discussion. Agreeableness reflects interpersonal tendencies to be altruistic and the belief that others will be so (McCrae and Costa, 2010). Low agreeableness relates specifically to SUD and not to other psychiatric disorders (Kotov et al., 2010). A reduced motivation and/or ability to maintain positive relations with others might result from the high degree of childhood trauma (Heffernan et al., 2000) and comorbidity of AD/HD (Frodl, 2010) found in patients with SUD. It is also possible that low Agreeableness is adaptive within the subculture of people using drugs.

The meta-analysis by Kotov et al. (2010) concluded that lower scores on Extraversion appeared to be associated with SUD, but the effect size was small. In line with this, a Norwegian opioid dependence population showed low scores on Extraversion (Nordvik and Kornør, 2007). However, studies of other SUD- and drug-using populations have found elevated scores on Extraversion (Delic et al., 2017; Erevik et al., 2017b; Raketic et al., 2017), and it’s facet excitement-seeking (Ruiz et al., 2003; Randhawa, 2018). The divergence in findings on Extraversion among individuals with SUD might partly result from diverse personality inventories across studies or reflect an essential difference in personality between different subgroups of SUD, e.g., type of substance used, frequency of intake/severity, and gender. Fewer studies have reported divergent findings on Openness, but elevations in Openness have been found among student cannabis users (Terracciano et al., 2008; Erevik et al., 2017b) and low levels on Openness among students with problematic alcohol patterns (Erevik et al., 2017a). Further, elevated and lower scores on different facets of Openness in SUDs have been found (Randhawa, 2018), while others have not found differences between individuals with SUD and controls on the facets of Openness (Raketic et al., 2017).

Gender and age differences in personality might explain inconsistencies across previously studied SUD populations. In addition, knowledge regarding gender and age differences in personality among SUD patients may help individualize treatment plans. We have not identified studies specifically investigating differences between men and women and different age groups regarding personality traits within SUD samples. More men than women use drugs (United Nations, 2021), and more men are treated with specialized addictions treatments (Helsedirektoratet, 2019). This indicates that drug problems are more atypical for women than men. Thus, women might be speculated to have more deviant personalities than men within SUD-samples. Personality traits change from adolescence to adulthood following a maturation principle with increased mean levels of Agreeableness and Conscientiousness and decreasing Neuroticism (Allemand et al., 2008; Bleidorn and Hopwood, 2019). This is the opposite of the low *Stability* SUD-profile described above. Mean-level changes indicate that we should find cross-sectional age differences within SUD-cohorts corresponding to the maturation principle. In contrast, if the typical SUD-profile to some degree is a result of a life with SUD, one might expect the opposite; the longer time with addiction, the more typical SUD-personality the person might get.

The present study investigates personality scores in a treatment-seeking SUD cohort, including age-and gender-related differences in personality scores.

## Materials and methods

### Participants

The present study (*n* = 123) is a substudy of the ongoing Stavanger Study of Trajectories of Addiction (STAYER, *N* = 208; Hagen et al., 2016), a 10-year longitudinal cohort study. STAYER recruited patients starting a new treatment sequence at ten outpatient and residential specialized treatment facilities for addiction disorders within the Stavanger University Hospital catchment area, Norway from March 13, 2012 to December 2, 2016. To access specialized treatment for addictions within the Norwegian public health service, patients must fulfill the criteria for a diagnosis of F1x.1 harmful use, F1x.2 dependency syndrome or F63.0 Pathological gambling, as defined by the International Classification of Diseases, 10th edition (ICD-10; World Health Organization, 1992). Patients stated at least 2 weeks of sobriety at baseline assessment. The data used in the current study stem from the baseline assessment in STAYER or in some instances the 3-or 6-month follow-up for participants who completed the NEO-PI-R at one of these follow-ups instead of at baseline. All participants in STAYER were invited to complete the NEO-PI-R, but we only included participants with SUD in this study, i.e., not patients with alcohol use disorder or pathological gambling. Two research assistants collected the data. Participants received a gift card with NOK 400 (∼40€) in compensation for their time at baseline testing and NOK 200 (∼20€) for the follow ups. STAYER was approved by the Regional Ethical Committee (REK 2011/1877).

### Measures

Clinical and social demographic data including year and country of birth, whether they had a permanent residence (yes/no), debut age of substance use (in years), and education level were registered at baseline. The research assistants ticked off gender (man/woman).

#### Personality

The NEO Personality Inventory–Revised (NEO-PI-R; Costa and McCrea, 1992) is a 240-item questionnaire designed to measure FFM traits, including Neuroticism, Extraversion, Openness, Agreeableness and Conscientiousness. Each trait consists of six facets, and eight items measure each facet. Each item consists of a statement concerning typical behavior, feelings, or cognitions to which the respondents are asked to indicate the degree to which the statements apply. Items are answered on a five-point Likert scale ranging from “strongly disagree” to “strongly agree.” Written self-report is the standard way of responding, but participants in the present study could also choose to reply vocally in response to being read the questions. This is a general adjustment applied to all the questionnaires included in the STAYER study, along with several other strategies to increase participation and maintain high retention rates (Svendsen et al., 2017). Because of the patients’ expected low literacy and education level, we believed that allowing vocal answering would increase participation, limit attrition and decrease the likelihood of random responding. Raw scores on the NEO-PI-R are transformed into *T*-scores in which a *T*-score of 50 reflects the norm sample’s mean and the standard deviation is 10 *T*-score points. In the current study, we had access to the participants’ *T*-scores as compared to the Norwegian gender specific norms on traits and facets (i.e., not their raw scores). The Norwegian translation of the NEO-PI-R has been validated in several samples (Martinsen et al., 2003, 2011; Martinsen, 2017).

### Statistics

Comparison of the *T*-scores between our sample and the Norwegian norm sample were conducted using an independent sample *t*-tests, with pooled standard deviation for unequal sample sizes. When investigating differences in personality traits within our sample (age-and gender differences, *t*-tests and chi-square tests, respectively). *P*-values < 0.01 were considered statistically significant for these tests. We considered moderate and large effect sizes, Cohen’s *d* ≥ 0.5 or Pearson’s *r* ≥ ± 0.3, to be of potential clinical relevance (Cohen, 1988). All tests were two-tailed.

IBM SPSS Statistics 26 was used for statistical analyses.

## Results

A total of 151 STAYER participants completed the NEO-PI-R; most of them at the 3-month follow-up (*n* = 143), and a few at baseline (*n* = 3) and 6-month follow-up (*n* = 5). Despite three opportunities and encouragement from the research assistants, 57 of the STAYER-patients did not participate or complete the NEO-PI-R. Some of these did start completion of the NEO-PI-R but did not finish; hence, these data were not included. Of the 151 NEO-PI-R responders, we only included responders with SUD in this study. A total of 28 responders did not suffer from SUD, but from alcohol use disorder (*n* = 20) or pathological gambling (*n* = 8). Hence, 123 responders were included in the further analyses. Of the 123 included responders, 117 completed the NEO-PI-R at the 3-month follow-up, 2 at baseline, and 4 at the 6-month follow-up.

There were no statistically significant (*p* < 0.05) differences between the included participants (*n* = 123) and the non-responders with SUD (*n* = 41) concerning the two age of substance use debut, number of earlier treatment attempts, whether they had completed any education after mandatory school, grades at school or years of working experience. Table 1 display sociodemographic characteristics of the included participants.

**TABLE 1:** Sociodemographic characteristics of included participants.

Variable	SUD *(n* = 123)
Mean age in years *(SD)*	27 (7)
Men (%)	63.4
Born in Norway (%)	91
Debut age substance use *(SD)*	13.2 (2.3)
Permanent residence (%)	57
No education^a^ (%)	61

Characteristics as registered at the time of inclusion. SUD, substance use disorder.

^a^No completed formal education after 9 or 10 years of mandatory school.

Internal consistency between the facet scores was high for Neuroticism (*α*^C^** = 0.85) and Conscientiousness (*α*^C^** = 0.85), while it was lower for Agreeableness (*α*^C^** = 0.76), Extraversion (α*^C^* = 0.73) and Openness (α*^C^* = 0.72). Internal consistency at the item level was impossible to evaluate since only facet and factor score data were available for this study.

### Personality traits in this cohort

The SUD-group scored higher (*p* < 0.01) on Neuroticism and lower on Extraversion, Openness, Agreeableness, and Conscientiousness compared to the Norwegian norms (see Table 2). The effect sizes of these differences were large for Neuroticism and Conscientiousness and moderate for Agreeableness and Extraversion. Our cohort’s statistically significant lower score on Openness had a small effect size.

**TABLE 2:** NEO-PI-R in our sample (*n* = 123) compared with the Norwegian norm sample (*n* = 3,521).

Factor (Chronbach’s alpha)/Facet	M (SD)	*P*-value	Cohen’s *d*
N: Neuroticism (0.85)	60.3 (9.8)	**<0.001**	**1.05**
E: Extraversion (0.73)	44.7 (8.9)	**<0.001**	**0.56**
O: Openness (0.72)	46.4 (9.5)	**<0.001**	0.37
A: Agreeableness (0.76)	43.3 (11.1)	**<0.001**	**0.63**
C: Conscientiousness (0.85)	41.7 (10.1)	**<0.001**	**0.83**
N1: Anxiety	57.9 (10.2)	**<0.001**	**0.78**
N2: Angry hostility	58.8 (9.9)	**<0.001**	**0.88**
N3: Depression	59.7 (9.5)	**<0.001**	**0.99**
N4: Self-consciousness	57.4 (9.7)	**<0.001**	**0.75**
N5: Impulsiveness	53.8 (9.8)	**<0.001**	0.38
N6: Vulnerability	59.2 (10.0)	**<0.001**	**0.92**
E1: Warmth	44.6 (10.2)	**<0.001**	**0.53**
E2: Gregariousness	42.4 (11.1)	**<0.001**	**0.72**
E3: Assertiveness	45.7 (9.9)	**<0.001**	0.43
E4: Activity	46.5 (9.0)	**<0.001**	0.37
E5: Excitement-seeking	53.7 (8.4)	**<0.001**	0.40
E6: Positive emotions	45.2 (9.1)	**<0.001**	**0.50**
O1: Fantasy	49.0 (8.3)	0.18	0.11
O2: Aesthetics	48.8 (9.8)	0.20	0.12
O3: Feelings	48.3 (9.7)	0.06	0.17
O4: Actions	46.3 (9.1)	**<0.001**	0.39
O5: Ideas	47.5 (11.1)	0.02	0.24
O6: Values	44.3 (8.2)	**<0.001**	**0.62**
A1: Trust	41.5 (10.0)	**<0.001**	**0.85**
A2: Straightforwardness	42.2 (11.8)	**<0.001**	**0.71**
A3: Altruism	46.2 (10.9)	**<0.001**	0.36
A4: Compliance	46.5 (11.2)	**<0.001**	0.33
A5: Modesty	50.5 (11.0)	0.59	0.05
A6: Tender-mindedness	47.3 (10.5)	0.01	0.23
C1: Competence	39.5 (11.1)	**<0.001**	**0.99**
C2: Order	49.5 (8.7)	0.51	0.05
C3: Dutifulness	40.8 (10.7)	**<0.001**	**0.89**
C4: Achievement striving	46.2 (10.7)	**<0.001**	0.37
C5: Self-Discipline	43.6 (9.2)	**<0.001**	**0.67**
C6: Deliberation	42.7 (9.3)	**<0.001**	**0.76**

Two-sample *t*-test, with pooled standard deviation for unequal sample sizes. For information about the Norwegian norm sample, see Martinsen (2017), Table 5A. *P*-value < 0.01 in bold. Effect size: Cohen’s *d* ≥ 0.5 in bold.

All facets of Neuroticism were statistically significantly elevated, and most of these differences showed a moderate or large effect size. The high scores on *N3: Depression*, *N6: Vulnerability*, and *N2: Angry Hostility* had large effect sizes. The only facet of Neuroticism that did not show a difference of moderate or high effect size was *N5: Impulsiveness*.

Five of the six Extraversion facets were significantly lower than the norm group, and the sixth was significantly higher. The low scores on *E2: Gregariousness*, *E1: Warmth*, and *E6: Positive Emotions* had moderate effect sizes. In contrast, the facet *E5: Excitement-Seeking* was elevated, but this significantly elevated score had only a small effect size.

The Openness facets *O4: Actions* and *O6: Values* were low compared to the norm group, but only the latter showed a moderate effect size.

Four Agreeableness facets were lowered, with a large effect size on *A1: Trust* and moderate effect size on *A2: Straightforwardness*.

Within the Conscientiousness facets, it was only *C2: Order* that was not significantly low. The low scores on *C1: Competence* and *C3: Dutifulness* had large effect sizes, while the low scores on *C6: Deliberation* and *C5: Self-Discipline* had moderate effect sizes.

We investigated whether personality traits varied within our cohort depending on age and gender. Results are shown in Table 3. Age correlated with Agreeableness and the effect size of this correlation was medium The facets *A2: Straightforwardness* (*r* = 0.33) and *A1: Trust* (*r* = 0.28) showed significant correlations with age. Further, the Extraversion facet *E5: Excitement-Seeking* showed a negative correlation with age (*r* = −0.27). No other statistically significant correlations were found between age and trait or facet scores.

**TABLE 3:** NEO personality inventory–revised (NEO-PI-R) correlated with age and gender among patients with substance use disorder (SUD) (*n* = 123).

Factor/Facets	Age		Gender			
	*r*	*P*-value	Men (*n* = 78)	Women (*n* = 45)	*P*-value	*d*
N: Neuroticism	0.00	0.99	61.7	57.9	0.04	0.38
E: Extroversion	–0.04	0.64	44.5	45.1	0.72	0.07
O: Openness	–0.02	0.81	47.5	44.5	0.09	0.31
A: Agreeableness	**0.32**	**<0.001**	43.7	42.5	0.58	0.11
C: Conscientiousness	0.06	0.48	41.2	42.6	0.47	0.14
N1: Anxiety	0.10	0.27	59.6	54.8	0.01	0.48
N2: Angry hostility	–0.12	0.19	59.8	57.1	0.15	0.27
N3: Depression	0.05	0.62	61.2	57.0	0.02	0.44
N4: Self-consciousness	–0.02	0.81	57.9	56.6	0.48	0.13
N5: Impulsiveness	–0.07	0.46	54.7	52.3	0.19	0.24
N6: Vulnerability	0.02	0.79	60.5	57.0	0.06	0.35
E1: Warmth	0.11	0.22	45.4	43.2	0.25	0.21
E2: Gregariousness	–0.03	0.79	42.5	42.1	0.83	0.04
E3: Assertiveness	–0.04	0.69	45.2	46.5	0.47	0.13
E4: Activity	0.03	0.76	46.5	46.5	0.99	0
E5: Excitement-seeking	–0.27	**0.003**	52.4	55.8	0.03	0.42
E6: Positive emotions	0.02	0.81	45.8	44.0	0.29	0.20
O1: Fantasy	–0.07	0.47	50.1	46.7	0.02	0.44
O2: Aesthetics	–0.09	0.31	49.9	47.1	0.13	0.28
O3: Feelings	0.15	0.10	49.6	46.1	0.08	0.35
O4: Actions	0.10	0.27	47.5	44.4	0.07	0.34
O5: Ideas	–0.06	0.48	47.8	47.0	0.70	0.07
O6: Values	–0.03	0.74	43.6	45.7	0.17	0.26
A1: Trust	0.28	**0.002**	41.6	41.4	0.89	0.02
A2: Straightforwardness	**0.33**	**<0.001**	42.9	41.0	0.39	0.16
A3: Altruism	0.12	0.19	46.6	45.5	0.61	0.10
A4: Compliance	0.20	0.03	46.9	45.7	0.58	0.11
A5: Modesty	0.17	0.06	50.4	50.8	0.85	0.04
A6: Tender-mindedness	0.19	0.04	47.5	47.0	0.80	0.05
C1: Competence	0.03	0.78	39.2	40.1	0.67	0.08
C2: Order	0.00	1	48.4	51.3	0.07	0.33
C3: Dutifulness	0.06	0.54	40.0	42.0	0.32	0.19
C4: Achievement striving	0.04	0.69	45.6	47.3	0.39	0.16
C5: Self-discipline	0.09	0.31	43.4	43.9	0.78	0.05
C6: Deliberation	0.04	0.66	42.2	43.4	0.49	0.13

PSUD = Polysubstance use disorder. Significance level < 0.01 in bold. Effect size d ≥ 0.05 or r ≥ +/− 0.03 in bold.

There were no statistically significant (*p* < 01) gender differences within our sample.

## Discussion

This SUD cohort scored high on Neuroticism, and low on Conscientiousness, Agreeableness, Extraversion and Openness compared to the Norwegian norm group. Although the Extraversion trait was moderately low compared to the norm group, its facet *E5: Excitement-Seeking* was significantly above average. Age showed a positive correlation with Agreeableness and specifically its facets *A2: Straightforwardness* and *A1: Trust*, and an inverse correlation with *E5: Excitement-Seeking.* Compared to their respective norm groups, there were no significant differences between the genders.

High Neuroticism and low Extraversion, Openness, Agreeableness and Conscientiousness correspond to a low score on the general factor of personality, *Big One*, and the two metatraits *Stability* and *Flexibility* (Musek, 2007). Such profiles are associated with less favorable outcomes, e.g., in terms of health, work, and education (Vedel, 2014; McAdams et al., 2019). Further, extremes of the five traits in the direction found in our cohort, corresponds to four of the dysfunctional traits in the alternative model of personality disorders in the Diagnostic and Statistical Manual of Mental Disorder, Fifth Edition (DSM-V): Negative affect (∼high Neuroticism), Detachment (∼low Extraversion), Antagonism (∼low Agreeableness), Disinhibition (∼low Conscientiousness), but not Psychoticism (∼high Openness; American Psychiatric Association, 2013). Indeed, one might speculate that the finding of a low score on the *Big One* reflects the high degree of comorbid personality disorders found in SUD patients (Verheul, 2001; Arnevik et al., 2019).

Although our cohort diverged significantly from the Norwegian norms on all five traits, the effect sizes were largest on the SUD-profile traits Neuroticism, Conscientiousness and Agreeableness. It was also medium for Extraversion. A SUD-profile equals a low score on the metatrait *Stability* (DeYoung and Allen, 2019). There are several possible explanations for low *Stability* in individuals with SUD. There might be common predictors of low *Stability* and SUD, e.g., childhood trauma (Spinhoven et al., 2016; Zhang et al., 2020); persons low in *Stability* might be more susceptible to SUD, and/or the use of substances and living within the SUD-culture might contribute to lower scores on Stability. Furthermore, low *Stability* in SUD-patients might represent absent or delayed maturation as these traits generally increase from the end of adolescence and into adulthood (Furnham and Cheng, 2019). While their peers often struggle with identity-issues in their teens (Kroger et al., 2010), this coincides with when patients with SUD typically start using drugs (Baldwin et al., 2013, see also Table 1), perhaps restricting normal personality development. Put together, there are probably multiple and coexisting causal relationships between the *Stability* traits and SUD. Unfortunately, the current study does not contribute to inform on the causality between personality traits and SUD. However, low Stability has several potential clinical implications for patients with SUD.

Neuroticism is especially high in our cohort, with a large or moderate effect size on the differences from the norm sample on all facets except on *N6: Impulsiveness*. The surprising finding that *N6: Impulsiveness* was only slightly above the norms, might reflect that our cohort consisted of patients who had used substances for many years. Their substance use is no longer impulsive and driven by the initial responsiveness to substances, but is more compulsive and habitually, which is typical for addictive use (Baskin-Sommers and Foti, 2015). The differences between our sample and the norm group had large effect sizes for the factor score and its facets *N3: Depression, N6: Vulnerability*, and *N2: Angry Hostility*. This indicates a general tendency to experience negative affect, risk for psychiatric problems, high readiness to experience depressive affect and anger, and vulnerability to stress (McCrae and Costa, 2010). State effects of substance use or withdrawal symptoms are unlikely to fully explain these findings, considering that most participants completed the NEO-PI-R 3 months after baseline assessment. If high Neuroticism increases the risk of both SUD, symptom disorders (e.g., depression), and personality disorder, staying sober does not necessarily fix the latter two. From clinical practice with SUD patients, we know that many use substances to manage their mental disorders. When giving up this coping strategy, high Neuroticism indicates a need for integrated psychiatric and supportive treatment along with SUD treatment.

Conscientiousness is low in this SUD cohort, with a large effect size on the factor and its facets *C1: Competence* and *C3: Dutifulness*. This trait captures the will to achieve, and the low facet scores indicate low opinion of their own abilities and disobeying their ethical principles (McCrae and Costa, 2010). It follows that SUD patients might need extra support to achieve the goals they set in treatment. Although they desire change, e.g., sobriety, permanent residence, and work, they need help to stick to their goals and keep up the faith that change is achievable. Low Conscientiousness is associated with executive problems with flexibility and adapting to changing environmental contingencies and task demands (Fleming et al., 2016). For persons who have used substances since their early teenage years, becoming sober and living a life in sobriety, require adapting to new environments. Therapists and other service providers should be aware of these difficulties.

Agreeableness, the third factor of *Stability*, is low in our SUD cohort, with a moderate effect size. The lowest facet score is *A1: Trust*, with a large effect size, is associated with being cynical, skeptical and expecting others to be dishonest or dangerous (McCrae and Costa, 2010). This will probably challenge the alliance and compliance in therapy and other treatment settings.

The finding of low scores on both *Stability* and *Flexibility* probably resonates with characteristics specific to our SUD cohort. This cohort consisted of patients with a high degree of polysubstance use, an early debut age of substance use, little formal education, and a large minority without permanent residence (see Table 1). We believe this reflects a high degree of marginalization, a potential marker for more disadvantageous personality traits. Studies of presumably less marginalized SUD populations, e.g., population studies (Terracciano et al., 2008) or student populations (Erevik et al., 2017b), find less deviant and unfavorable personality traits. Differences in personality profiles across different SUD populations highlight that there is not one type of SUD population, and perhaps different personality profiles correspond to different SUD groups. This is in line with a study from Austria on patients in SUD treatment, where patients with alcohol use disorders had less deviant personality profiles than patients with poly-SUD (Lackner et al., 2013).

Earlier studies on SUD cohorts do not show consistent divergences from the norms on the traits Extraversion and Openness, which underlies the meta-trait *Plasticity* (Musek, 2007). More severe SUD, e.g., dependence, has been shown to have higher psychiatric comorbidity than less severe SUD, e.g., abuse (Goldstein et al., 2012). One might speculate that the significantly low scores on Extraversion and Openness in our sample are a consequence of many years of substance use, and hence a sign of severity. It might be hypothesized that severity of addiction would be associated with personality profiles farther from the norm, in the direction of less *Plasticity*. Although the findings on Extraversion and Openness were significant, it might be questioned how clinically relevant these findings are. Especially the relevance of Openness, since this finding only had a small effect size.

Extraversion has shown divergent results in different SUD cohorts: Elevated scores in young women with opioid addiction (Raketic et al., 2017) and in Norwegian students smoking cannabis (Erevik et al., 2017b), average scores in different SUD-groups (Terracciano et al., 2008), and low scores in samples with opioid dependency (Carter et al., 2001; Nordvik and Kornør, 2007) as well as in our sample. The meta-analysis by Kotov et al. (2010) concluded that SUD patients had low scores on Extraversion, but that the effect size was small. The low score on Extraversion in our cohort had a moderate effect size. While all the other facets were significantly lower in our cohort than in the norm group, the facet *E5: Excitement-Seeking* was slightly elevated. Our study is in line with Randhawa (2018), who found low Extraversion but high *E5: Excitement-Seeking* in a male SUD cohort in India. The discrepancy between high *E5: Excitement-Seeking* and the other facets in our cohort might contribute to explaining the inconsistent results regarding Extraversion across different SUD-populations. It might be that certain aspects of SUD, whether it be the direct drug effect or social and psychological factors associated with SUD, are associated with high and/or low scores on different facets of Extraversion. Further, an interaction between different facet-patterns on Extraversion and preferred drug is not implausible. Our cohort was dominated by patients with poly-SUD, a long career of drug use, and a certain severity considering them being patients in specialized treatment. This might be characteristics associated with low Extraversion.

The correlations we found between age and Agreeableness and *E5: Excitement-Seeking* in which older age was associated with higher Agreeableness and lower *E5: Excitement-Seeking* scores, are in accordance with the general mean-level development of traits in the general population (Furnham and Cheng, 2019). However, a short review by Furnham and Cheng (2019) indicates that Conscientiousness also increases and Extraversion and Neuroticism tend to decline over time, with most change happening before the age of 30. Our findings might indicate that this maturation effect is more robust for Agreeableness and *E5: Excitement-Seeking*, happening even during ongoing SUD, while the other traits perhaps do not change during ongoing SUD. It is possible that the general lack of maturation effects found in personality, except increasing Agreeableness, are results of life experiences that our SUD group to a lesser extent takes part in, e.g., work-life. Another potential explanation is that substance use or living in a subculture with substance users calls for continuous high levels of Neuroticism, hinders the development of Conscientiousness, and that the already low level of Extraversion does not allow for further decline. It is, however, important to note that the cross-sectional design of the current study implies that the observed age differences may be caused by factors other than maturation or the lack thereof. In addition, the effect sizes on age differences are small or close to small. A longitudinal approach to personality in SUD populations might enlighten these preliminary speculations. The few studies we have found following personality changes in SUD populations, have a maximum of 1 year between measure points. This is too short to conclude on maturation effects versus potential treatment effects.

Men are overrepresented in SUD populations (Goldstein et al., 2012; Lev-Ran et al., 2013; McHugh et al., 2018; United Nations, 2021). Since SUD among women is more uncommon than in men, one might expect that women with SUD have a more divergent personality compared to men with SUD. In contrast, men in our study tended to score farther from their norm group on Neuroticism (*d* = 0.38) and its facets *N1: Anxiety* and *N3: Depression* than women did However, this was only tendencies with small effect sizes, and not a statistically significant results. Women tended to score lower than their normgroup on *O1: Fantasy* while men had average scores on this facet. A previous study found gender differences of comorbid disorders in treatment-seeking substance users reflecting the gender differences in the general population, however, men had more affective disorders relative to women than would be expected from general population data (Brady and Randall, 1999). A Finnish study found that men with SUD reported more severe personality and emotional problems than women with SUD after controlling for education level, onset age of regular substance use, and polysubstance use (Höijer et al., 2021). This is in line with the tendency in our cohort, where men score high on *N3: Depression*. However, the tendency toward a gender difference compared to norm data in our cohort is not significant and probably of little clinical relevance. Brady and Randall (1999) found no gender differences in axis II diagnoses, matching our overall finding of few gender differences in normal personality traits. In an epidemiologic survey in the United States, Goldstein et al. (2012) found few gender differences in comorbid associations of specific SUDs with specific psychiatric disorders. While men are overrepresented in SUD, women are overrepresented in seeking psychiatric help (Kessler et al., 1981; Afifi, 2007). A possible explanation for the gender differences in personality traits that some have found in SUD cohorts, is that men typically master their anxiety and depression with more externalizing behavior such as substance use while women in general have a tendency toward internalizing emotional problems (United Nations, 2021). This explanation assumes the presence of these gender differences as precursors of SUD. Another possibility is that substance use affects these facets more strongly in men than women. In sum, the potential gender differences in personality traits within SUD cohorts seem to have little relevance compared to the general differences in personality traits between SUD cohorts and the general population.

### Strengths and limitations

STAYER planned to include a representative sample of treatment seeking patients with SUD. The inclusion of the full heterogeneity of SUD-patients contributes to high ecologic validity and increases the clinical relevance of the findings. However, the number of potential patients who did not consent to participate was not registered; hence, a possible non-response bias cannot be ruled out. On the other hand, we compared those STAYER participants who did with those who did not complete the NEO-PI-R, without finding differences.

While the heterogeneity of the cohort provides high ecologic validity, it leaves several questions unanswered. The questionnaire was completed at different times after inclusion, for the vast majority at the 3-month follow-up, but for some at baseline or at the 6-month follow-up. In addition, the period of abstinence before inclusion varied; it was a minimum of 2 weeks but could also be longer. The possible effect of time after abstinence is not possible to examine based on our data. Furthermore, comorbid mental disorders were not accounted for. Patients with SUD are known to have a high comorbidity with other mental illnesses (Goldstein et al., 2012).

The NEO-PI-R questionnaire is based on a self-report form. Dyslexia is a common learning difficulty, that is potentially overrepresented in people with SUD (Jhanjee, 2015), and most of the participants in this study chose to provide oral answers. This modification might have increased participation and the validity of the responses from persons with reading and writing difficulties. When giving oral response, the relationship to the research assistant might have reduced the risk of random responding, but it is also possible that it increased the risk of providing socially desirable responses.

It should be noted that the analyses of age differences are based on cross-sectional data. Hence, the differences found might be due to differences between the older and younger participants that are unrelated to age.

Whether it is sensible to measure personality in such an unstable group as treatment seeking patients with SUD, is a question embracing the construct validity of personality, and the intersection between personality traits and states (Fleeson, 2004). Personality traits are supposed to measure relatively stable individual differences (McAdams et al., 2019); nevertheless, the risk of added on state-artifacts (Roberts et al., 2017) when measuring personality traits during the initial phase of recovery cannot be ruled out. A few studies indicate a normalization of personality traits after recovery from AUD (Boulze et al., 2014; Betkowska-Korpala, 2015), but more research is needed to understand the longitudinal dynamics between SUD and personality.

## Data availability statement

The raw data supporting the conclusions of this article will be made available by the authors, without undue reservation.

## Ethics statement

The studies involving human participants were reviewed and approved by Regional Ethical Committee (REK 2011/1877). The patients/participants provided their written informed consent to participate in this study.

## Author contributions

EF, EE, EH, and AE planned this study. EF performed the literature review and preliminary data analysis and wrote the first draft, with EE as main supervisor. AU assisted on the data analysis. All authors contributed to the final draft and approved the submitted version.
